# Mouth Pain in Horses: Physiological Foundations, Behavioural Indices, Welfare Implications, and a Suggested Solution

**DOI:** 10.3390/ani10040572

**Published:** 2020-03-29

**Authors:** David J. Mellor

**Affiliations:** Animal Welfare Science and Bioethics Centre, School of Veterinary Science, Massey University, Palmerston North 4474, New Zealand; j.mellor@massey.ac.nz; Tel.: +64-21-390-855

**Keywords:** “bit blindness”, bitted to bit-free behaviour, conscious noxious experience, gum, tongue and lip pain, oral lesions, pain grimace, pain-induced breathlessness, anxiety and fear, remedial strategy

## Abstract

**Simple Summary:**

Mouth pain in horses, specifically that caused by bits, is evaluated as a significant welfare issue. The conscious experiences of pain generated within the body generally, its roles, and its assessment using behaviour, as well as the sensory functionality of the horse’s mouth, are outlined as background to a more detailed evaluation of mouth pain. Bit-induced mouth pain elicited by compression, laceration, inflammation, impeded blood flow, and the stretching of tissues is considered. Observable signs of mouth pain are behaviours that are present in bitted horses and absent or much less prevalent when they are bit-free. It is noted that many equestrians do not recognise that these behaviours indicate mouth pain, so that the magnitude of the problem is often underestimated. The negative experiences that are most responsible for welfare compromise include the pain itself, but also, related to this pain, potentially intense breathlessness, anxiety, and fear. Finally, a series of questions is proposed to clarify issues that are relevant to increasing the adoption of bit-free bridles in order to avoid bit-induced mouth pain.

**Abstract:**

A proposition addressed here is that, although bitted horses are viewed by many equestrians as being largely free of bit-related mouth pain, it seems likely that most behavioural signs of such pain are simply not recognised. Background information is provided on the following: the major features of pain generation and experience; cerebrocortical involvement in the conscious experience of pain by mammals; the numerous other subjective experiences mammals can have; adjunct physiological responses to pain; some general feature of behavioural responses to pain; and the neural bases of sensations generated within the mouth. Mouth pain in horses is then discussed. The areas considered exclude dental disease, but they include the stimulation of pain receptors by bits in the interdental space, the tongue, the commissures of the mouth, and the buccal mucosa. Compression, laceration, inflammation, impeded tissue blood flow, and tissue stretching are evaluated as noxious stimuli. The high pain sensitivity of the interdental space is described, as are likely increases in pain sensitivity due to repeated bit contact with bruises, cuts, tears, and/or ulcers wherever they are located in the mouth. Behavioural indices of mouth pain are then identified by contrasting the behaviours of horses when wearing bitted bridles, when changed from bitted to bit-free bridles, and when free-roaming unbitted in the wild. Observed indicative behaviours involve mouth movements, head-neck position, and facial expression (“pain face”), as well as characteristic body movements and gait. The welfare impacts of bit-related pain include the noxiousness of the pain itself as well as likely anxiety when anticipating the pain and fear whilst experiencing it, especially if the pain is severe. In addition, particular mouth behaviours impede airflow within the air passages of the upper respiratory system, effects that, in their turn, adversely affect the air passages in the lungs. Here, they increase airflow resistance and decrease alveolar gas exchange, giving rise to suffocating experiences of breathlessness. In addition, breathlessness is a likely consequence of the low jowl angles commonly maintained during dressage. If severe, as with pain, the prospect of breathlessness is likely to give rise to anxiety and the direct experience of breathlessness to fear. The related components of welfare compromise therefore likely involve pain, breathlessness, anxiety, and fear. Finally, a 12-point strategy is proposed to give greater impetus to a wider adoption of bit-free bridles in order to avoid bit-induced mouth pain.

## 1. Introduction

Things in plain sight can often be overlooked because they have established an ever-present familiarity in a context where competing ideas do not receive serious attention. Eventually, they become such an integral and essential part of current thinking that when other ideas are suggested they are confidently dismissed. However, when adherents to such widely accepted views are eventually faced with credible challenges, some become uneasy, defensive, and/or combative, whilst others seek to constructively explore how the new perspective may improve understanding. These well-recognised elements of the dynamics of change became apparent during the present author’s review of key publications that are relevant to the significance of mouth pain in horses. Although the equine veterinary and behavioural literature is replete with relevant reports, with relatively few exceptions (e.g., [1,2,3,4,5,6,7,8,9,10,11]), mouth pain itself does not appear to have received the direct attention it deserves [10]. 

The primary purpose of the present review is to rectify this situation. It begins with a detailed but brief account of the following: the key physiological characteristics of pain; evidence that pain is experienced consciously by mammals, including horses; the diversity of pain types generated widely throughout the body; and, generally, how pain may be assessed behaviourally (Section 2). Attention is then focused on the neural foundations of the rich intensity of sensations that are generated within the mouths of horses (Section 3). Thereafter, equine mouth pain is addressed directly, especially that caused by bits (Section 4). Particular attention is given to the following: the exceptional sensitivity of oral tissues to noxious stimulation; the major sites within the mouth where bit-related traumatic injuries occur; and compression, laceration, inflammation, impeded tissue blood flow, and tissue stretching as major stimuli that may cause mouth pain. Specific behavioural indices of bit-induced pain are then described by outlining the behaviours of horses ridden or driven while bitted and bit-free, and by comparing them with the behaviours of wild, free-roaming horses (Section 5). Negative animal welfare impacts of bit use are then shown to extend beyond bit-induced mouth pain to also include the interacting experiences of intense breathlessness, anxiety, and fear (Section 6). The review ends with a strategy designed to evaluate a potential solution to bit-induced mouth pain in horses by providing a coherent basis to expand the use of bit-free bridles (Section 7). 

## 2. General Features of Pain

### 2.1. Major Features of Pain Generation and Experience

A systematic description of the key attributes of pain was provided by the International Society for the Study of Pain in 1979 (Table 1) [12] and was subsequently paraphrased by others (e.g., [13,14,15]). Pain warns human beings (and other animals) that tissue damage might occur, is occurring, or has occurred, thereby enabling or eliciting immediate escape, withdrawal, or other behavioural responses (see Section 2.5). The experience of pain also alerts animals to opportunities for avoiding pain-causing circumstances in the future. Specific pain receptors (nociceptors) detect harmful or potentially harmful (i.e., noxious) stimuli that can cause tissue damage. Nociceptor-generated impulses are carried via their associated nerves to pain pathways in the spinal cord, or cranial nerves, and thence to the lower and higher centres of the brain where they are converted or transduced into conscious experiences of pain (i.e., perceived pain). The character of perceived pain varies according to the features of the nociceptive input (e.g., site, duration, intensity), and according to other factors that can affect the way the brain processes that input (e.g., prior experience, emotional state, individual variation). Pain is an unpleasant, sometimes an exceptionally unpleasant, subjective experience, which is usually linked to tissue damage. Across mammalian species, the pain detection apparatus is apparently equally sensitive, but pain tolerance may vary.

This characterisation of pain was later modified in order to be more applicable to non-human animals [16]: “*Animal pain is an aversive sensory and emotional experience representing an awareness by the animal of damage or threat to the integrity of its tissues; (note, that there may not be any damage); it changes the animal’s physiology and behaviour to reduce or avoid damage, to reduce the likelihood of recurrence and to promote recovery; non-functional pain occurs when the intensity or duration of the experience is not appropriate for the damage sustained (especially if none exists) and when physiological and behavioural responses are unsuccessful in alleviating it*.”

These attributes of pain apply to nociceptive inputs generated by the compression or stretching of sensitive tissues and by physically invasive injuries; other stimuli include those associated with impeded tissue blood flow (ischaemia), extreme heat or cold, corrosive agents, poisoning, and disease-induced pathology [13,14,15,17,18,19,20,21]. In line with these various causes of pain, it is noteworthy that at least 30 varieties of it have been identified [22]. However, the pain induced in the mouth by compression, bruising, laceration, ulceration, impeded tissue blood flow, and/or stretching of sensitive tissues has been emphasised here (Section 4). 

### 2.2. Cerebrocortical Involvement in the Conscious Experience of Pain in Mammals

Anthropomorphism, i.e., the attribution of human traits, emotions, or intentions to non-human entities, including animals, has been regarded by some commentators as a threat to the objectivity of scientific reasoning (e.g., [23,24]). Concerns about anthropomorphic reasoning have led, among other things, to skepticism regarding whether or not animals can experience pain in ways that cause them to suffer (e.g., [25,26]), and whether mammals other than human beings can be shown unequivocally to have any conscious experiences at all, including pain experience (e.g., [27,28]). Such concerns also underlie an apparent hesitation, reluctance, or extreme caution among some scientists to accept that animals can have a wide range of observable subjective experiences, including pain, which are of significance to their welfare (e.g., in horses [28,29]), despite a large body of supporting literature to the contrary (Section 2.3).

However, such views are not widely accepted by animal welfare and other scientists [15,20,30]. Instead, the majority hold that animals would consciously experience pain when it can be shown that coincident physiological and behavioural responses are indeed due to nociceptive inputs from areas of the body subject to noxious stimulation [13,14,15,20,21,30]. Such arguments rely on the cautious application of scientifically informed best judgement, which underlies conclusions that recognise a legitimate role for what has come to be known as “critical anthropomorphism” [30,31]. Moreover, evidence that the cerebral cortex of mammals has an integral role in the conscious experience of pain [32,33,34] further supports this reasoned approach.

It has long been known that changes in cerebrocortical function are aligned with alterations in the frequency components of cortical electrical activity, as recorded using an electroencephalogram (EEG) [35]. The statistical analyses of particular variables in EEG power spectra may be conducted using the well-established Fast Fourier Transformation method [36]. Specific changes in EEG power spectra have been shown to reflect alterations in the activity of the cerebral cortex associated with the conscious experience of pain [37]. Such spectral analyses of EEGs, used first in conscious human beings [37,38,39,40,41], were then applied to other animals (e.g., [42,43,44,45,46,47,48]) in studies of responses to the acute noxiousness of painful events. Examples of this include the immersion of an arm in ice water in humans, and surgery, castration, tail docking, or dehorning in animals. The majority of the animal studies have been conducted by utilising a “minimal anaesthesia model” [49] that enabled pain-free studies of specific EEG responses to otherwise consciously experienced painful stimuli. Each study, justified ethically, was aimed at improving understanding the life stages and situations where pain may be experienced, the mechanisms responsible for generating pain, and/or pain minimisation using pain-relieving agents. 

The model involves maintaining mammals on a stable, light plane of general anaesthesia; this allows particular cerebrocortical responses to different noxious stimuli to be detected by reducing the background variability in cerebrocortical electrical activity due to extraneous stimuli (for further details see [49,50]). To date, this technique has been used successfully to investigate responses to noxious sensory stimuli, as well as the effects of systemic analgesics and local anaesthetic nerve blockade, in a wide range of mammals, as follows:

Horses—castration [44] and the effects of systemic analgesia [45];

Red deer (*Cervus elaphus*)—velvet antler removal with and without nerve blockade [48];

Cattle—amputation dehorning with and without nerve blockade [51], the humaneness of slaughter [52,53,54,55];

Lambs—ontological changes in responses to castration [47,56];

Pigs—ontological changes in responses to tail docking [57], the noxious effects of castration with and without nerve blockade [46], the humaneness of intraperitoneal sodium pentobarbital injection as a killing method [58], postnatal changes in responses to noxious stimulation [59];

Tammar wallabies (*Macropus eugenii eugenii*)—ontological changes in responses to toe clamping [60];

Rats—ontological changes in responses to tail clamping [61], the effects of noxious thermal, mechanical, and electrical stimuli [62], the humaneness of decapitation as a killing method [63], the efficacy of systemic analgesics [64]; 

Dogs—the effects of novel analgesics [65].

These observations on cerebrocortical responses support the conclusion that, depending on the circumstances, noxious sensory inputs, due to nociceptor stimulation, can result in a wide range of mammals, including horses, having conscious experiences of pain. 

### 2.3. Terrestrial Mammals Can Have Numerous Other Subjective Experiences in Addition to Pain

In addition to the many varieties of pain [22], there are numerous other subjective sensations, feelings, and emotions, collectively known as ‘affects’ [66], which animals may experience consciously. Evidence for this has been provided during the last 15–20 years by numerous studies of the brain processing of specific sensory inputs that lead to the generation of different affects (for references, see [30,67,68,69,70,71,72,73]. There are affects related mainly to the internal state of the animal, which include breathlessness, thirst, hunger, nausea, dizziness, debility, weakness, and sickness, as well as pain. Another group of affects mainly reflects the animal’s perception of its external circumstances. These affects may be negative, such as anxiety, fear, panic, frustration, anger, helplessness, loneliness, boredom, and depression, or, when animals utilise opportunities to have rewarding experiences, the affects may be positive and include various forms of comfort, pleasure, interest, confidence, and a sense of being in control. The major conclusion from these affective neuroscience observations is that many mammals, including horses, are demonstrably capable of consciously experiencing a wide range of positive and negative subjective sensations, feelings, and emotions, including pain in its various noxious forms. 

### 2.4. Adjunct Physiological Responses to Pain-Inducing Stimuli

There are other physiological changes elicited by potentially painful stimuli in mammals, many of which involve the sympathetic nervous system (SNS) and the hypothalamic–pituitary–adrenocortical axis (the HPA axis). Detailed descriptions of these responses and of their usefulness as indirect indices of pain and the effectiveness of analgesics have been provided elsewhere (e.g., [14,17,18,19,20,21,28,30,74,75,76,77,78,79,80,81]). Briefly, SNS responses include changes in the circulating concentrations of adrenaline and noradrenaline, as well as related changes in heart rate, blood pressure, respiratory rate, and whole body or regional temperature; HPA responses have usually focused on elevations in circulating glucocorticoid concentrations. SNS and HPA responses have been studied extensively in cattle, sheep, goats, pigs, horses, and other mammals. Although these physiological changes have been well validated as responses to nociceptor stimulation, they may also capture simultaneous impacts of other negative states [13,17,28,82], for example, fear, which may be elicited directly by the pain itself, or which may be due to frightening aspects of the circumstances in which the pain is experienced (see Section 6), for example, the presence of predators or other threats.

### 2.5. General Behavioural Responses to Pain

Behaviour is perhaps the most often used indicator of when animals are in pain. Typically, they exhibit changes from normal day-to-day behaviours reflected in appearance, demeanour, posture, gait, activity/inactivity, vocalisation/silence, interactions with other animals (including people), and reactivity to handling. Moreover, animals may “guard” or protect the painful site [14,20,21,83]. Additional signs include a decline in or an absence of behaviours that the animals are usually highly motivated to perform, such as playing, grooming, eating, and sleeping [15,20,71]. The administration of local anaesthetics and/or systemic analgesics also partially or completely restores normal behaviours, or diminishes specific pain-related behaviours [14,15,17,21,74,76,77,80,83,84,85,86,87,88,89].

Building on the longstanding application of facial expression to the assessment of pain in human beings, interest in utilising this approach to assess the presence, absence, intensity, and/or duration of pain in other mammals has burgeoned in recent years (see [15] for a detailed review). Facial expression is assessed by using a coding scheme that measures or records changes in the disposition of individual “action units” of the face. Taken together, these allow distinct facial expressions to be identified, albeit with species-specific features, when animals are and are not experiencing pain; they may also offer the potential for scaling the intensity of pain when present. To date, “grimace scales” or “pain faces” have been described for mice, rats, rabbits, ferrets, cats, piglets, lambs, sheep, and horses [15]. It is noteworthy for the present purposes that scales for horses have recently received significant attention [90,91,92,93,94,95,96,97]; however, note the caveat that the prevalence of observable pain-related behaviours tend to decrease when caregivers are with the horse [98]. After evaluating the strengths, weaknesses, validation, reliability, and practicality of all of these mammalian scales, McLennan and colleagues concluded that:

“*There is good evidence that facial expression can be a useful, valid and reliable tool for recognising and evaluating pain in humans and other animals. Both the sensory and emotional components of pain have been demonstrated to affect facial expression, which thus gives a true representation of the affective state of the animal. Many of the mammalian species studied to date have similar facial expression responses to pain*.”

[15]

They then provided advice on further studies that would enhance the value of these scales for pain assessment. Finally, it is noted here that underlying all such scales is the explicit understanding that the mammals to which they apply can and do consciously experience pain, and that pain is affectively noxious [15].

### 2.6. Summing up

The above general observations focus on terrestrial mammals. They refer to the numerous different types of pain generated by a multiplicity of causes that impinge on organs and tissues throughout the body. It is apparent that pain, among a variety of other negative affects, is experienced consciously and is often unpleasant, sometimes intensely unpleasant. Indices of different pains may be physiological and/or behavioural. However, the utility of such indices depends heavily upon accessing contextual information in order to improve the capability to identify specific types of pain and to discriminate that pain from other factors, such as fear. Accordingly, after briefly considering the wide range of sensations generated within the oral cavity (Section 3), the focus here will shift specifically to mouth pain in horses, its potential causes, and its recognition (Section 4).

## 3. Sensations Generated within the Mouth

The tissues of the mouth are among the most richly innervated of any in the body regarding the number and variety of sensory receptors they contain [99]. Excluding taste, these receptors sensitively detect mechanical events (touch), thermal events (hot, cold, warm) and noxious events (pain). The associated impulse traffic is transmitted via the trigeminal nerve to lower regions of the brain and then upward to specific areas of the sensory cortex [99]. Here, the impulses are processed into particular conscious sensations in ways that also identify their sites of origin, for example, the gums, palate, other areas of the oral mucosa, the teeth, tongue, masticatory muscles and/or facial skin [99]. Taken together, these sensations provide information about the state and structure of the mouth itself and objects within the mouth, including foreign objects. Moreover, the richness of this sensory innervation means that stimulation of oral tissues generates bodily experiences that can be among the most intense possible [99].

### 3.1. Tissues Supplied by the Trigeminal Nerve

The name “trigeminal” is derived from the Latin word “tria”, meaning three, and “geminus”, meaning twin. Thus, although commonly referred to in the singular form as “the trigeminal nerve”, it in fact consists of a pair of nerves, one running to the tissues on the right and the other to the tissues on the left side of the head. The trigeminal is the fifth of 12 pairs of cranial nerves which emerge directly from the brain [100,101,102]. By convention they are labelled with the Roman numerals I–XII, the trigeminal being V. The paired trigeminal nerves each have three branches, designated V1, V2, and V3. As noted, these carry touch-, temperature- and/or nociceptor-related information to the brain: V1 from the eye (cornea, ciliary body, lacrimal gland, conjunctiva), the skin of the upper eyelid, forehead and nose, and the mucous membranes of the nasal cavity; V2 from the lower eyelid, side of the face, nose, upper lip, upper gum, and upper teeth; and V3 from the lower lip and chin, the jaw, lower gum and mandibular teeth, the mucous membranes of the rostral two-thirds of the tongue, some of the muscles used for chewing, and some parts of the ear. V3 also incorporates motor fibres that innervate the muscles of mastication, the ventral surface of the oral cavity, and the palate.

### 3.2. Tissues Supplied by the Facial Nerve

The facial nerve (VII) has both sensory and motor functions [100,101,103]. The sensory fibres of the facial nerve innervate the rostral two-thirds of the tongue, an area also supplied with trigeminal V3 fibres. However, VII is mainly a motor nerve. Its fibres innervate the ear canal, salivary glands (parasympathetic control), lacrimal glands, nasal cavity, palate, and muscles of facial expression. The facial muscles are superficial, flat and thin, originate from bony structures of the skull and then radiate out to adjacent areas of the skin. Facial expressions that constitute pain-induced grimaces (see Section 2.5) or “pain faces” (e.g., [94]), reflect nociceptive inputs via the trigeminal nerve (V), as well as inputs from elsewhere in the body carried in the spinal nociceptive pathways [15]. 

## 4. Mouth Pain in Horses

The exceptional sensitivity of oral tissues to noxious stimulation [99] highlights the importance of understanding the various ways common riding or driving practices would stimulate oral nociceptors sufficiently to cause horses significant pain. The types of stimuli considered here are compression, laceration, inflammation, impeded tissue blood flow, and tissue stretching, and the practices considered are the use of bits and, briefly, tongue ties. Disease-related dental pain is not considered.

### 4.1. Bit-Induced Nociceptor Stimulation and Pain

#### 4.1.1. The Interdental Space

Bridles are usually adjusted so that the bit is in contact with a largely tooth-free segment of the gums on each side of the mandible, i.e., behind the incisors and in front of the premolars in the so-called “interdental space” [7,104,105,106]. The gums are modified periosteum, i.e., the membrane that surrounds bone, and are richly supplied with nociceptors [99,107]. Accordingly, rein tension transmitted as bit pressure applied to the mandibular gums can readily generate intense pain, especially as the pressure per unit area of direct bit–gum contact is amplified by the round cross-section of the bit and the usually narrow upper edge of the interdental mandible [7,11,104,108].

The magnitude of this amplification can be estimated by utilising the following information.

(1). The established relationship between tension (T, units N), mass (m, units kg) and gravitational acceleration (g = 9.8 metres/sec^2^), which is “T = mg” or “T = 9.8 m” [109].
(2). Known rein tensions in various situations. Examples include zero N (Newtons) with a loose rein, maxima of 51 to 166 N, and mean values that ranged from 9 to 59 N [110,111,112,113,114,115].
(3). An estimated area of bit–gum contact on the interdental space (CA_bg_) of 0.387 cm^2^ [2], which is equivalent to a 6.22 × 6.22 mm square.

The mass equivalent (kg) of rein tension (N) may be calculated using a different form of the above equation, namely “m = T / 9.8”, and the mass per unit cross-sectional area (kg/cm^2^) using “m / 0.387”. Thus, the mass equivalents of the above values are a minimum of zero kg, a range of maxima of 5.2 to 16.9 kg, and a range of overall mean values of 0.9 to 6.0 kg, respectively. The related figures for mass per unit area are zero kg/cm^2^ for the minimum, 13.4 to 43.7 kg/cm^2^ for the range of maximum values, and 2.3 to 15.5 kg/cm^2^ for the range of overall mean values. The estimated amplification factor is 2.58. Apart from the “loose rein” minimum, and a report of estimated mean bit pressures that were mostly between 0.93 and 1.1 kg/cm^2^ [116], most of the above bit pressures would be painful, some of them exceptionally so. It is therefore of interest that, with one exception [115], bit-induced pain was not mentioned in any of the above papers on rein tension [110,111,112,113,114,116,117].

**Figure 1:**
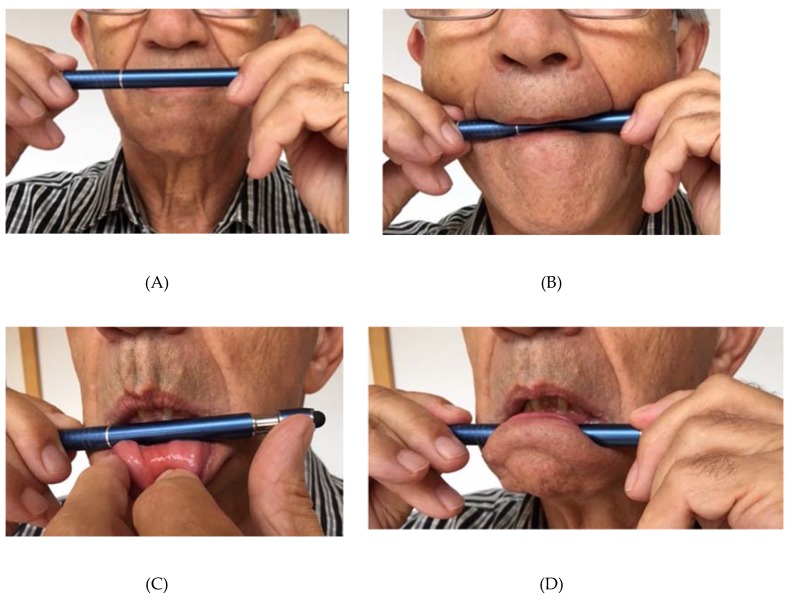
The ‘Mellor pen test.” This simulates bit pressure applied to the gums of the interdental space of the horse. Gums are exquisitely sensitive to painful stimuli, including compression. Rein tension transferred to the bit in contact with the gums of the interdental space causes pain. (**A**) Position 1: Hold the pen in front of your mouth; (**B**) Position 2: Open your mouth, place the pen where the upper and lower lips meet on each side, and then push the pen towards the back of your throat. No gum contact, no significant pain; (**C**) Position 3a: Roll your bottom lip down and locate the pen on your gum, below your central incisors; (**D**) Position 3b: Now release your lip and with both hands holding the pen, apply compressive pressure to your gum, carefully increasing the pressure in steps from very low until the pain is too intense to continue. How much compression-induced pain could you stand?

Readers may gain a personal insight into the likely intensity of such pain by conducting on themselves what has come to be known as the “Mellor pen-test”. This test is intended to simulate the compressive effects of bit pressure applied to the gums of the interdental space of a horse. It involves applying pressure to the barrel of a pen placed against the gums below the front incisor teeth of the lower jaw (Figure 1). In common with the experiences of audiences totaling at least 450 addressed by the author to date (e.g., see [118]), it is anticipated that the vast majority of readers will find that intense pain may be generated by low pressures. 

As a further exercise, first access a set of top-loading kitchen scales for weighing up to at least 3kg. With an index finger pointing down vertically, place its *tip* (not the distal fingerprint surface) on the weighing tray so that the bone of the terminal phalanx bears most of the pressure; and then press directly downward to hold the scale readings successively at 1, 2 and 3 kg, taking a break between each level. Bearing in mind that the fingertip is much less immediately susceptible to pain-inducing pressure than are the exquisitely sensitive gums, readers should note how long they can maintain these scale readings before pain compels withdrawal. Now compare these scale levels with the values for bit pressure per unit area (kg/cm^2^) given above and note that 3 kg/cm^2^ is considerably less than most of them.

Finally, the reader may also wish to imagine lying flat on their back on a raised platform with sufficient space under it to suspend a small carry-on aircraft flight bag. The bag is attached to light reins fixed to each end of a metal bit located, as with the “Mellor pen test”, on the mandibular gums below the front incisor teeth (Figure 1). Now imagine that the weight of the bag is increased from 2 up to a 7 kg carry-on maximum. Then, imagine that the weight is increased progressively to a 20 kg maximum for stowed luggage, noting that this is less than half the figure of 43.7 kg/cm^2^ derived from the 166 N maximum rein tension referred to above. 

It is anticipated that the combined results of these three exercises will speak for themselves.

The mandibular periostitis (bone spur formation) observed in the interdental space of horses wearing bitted bridles and its absence or virtual absence in free-roaming or feral equids, when taken together, provide evidence of significant traumatic impacts of bit use. Three postmortem studies of equid mandibles illustrate this: (1) interdental bone spurs were found in ~88% of 32 working horses but there were none in 28 Przewalski horses [119]; (2) interdental space roughness was reported in 48% of 87 Warmbloods or trotters, in 25% of eight donkeys, but only in 7% of 68 zebra [108]; and (3) spurs were observed in ~61% of mandibles from 66 domestic horses, but none were seen in 12 feral and Przewalski horses [7]. In addition, live assessment of oral lesions revealed that 28–30% of 50 polo ponies and 50 racehorses had interdental bone spurs, which were generally more severe in the racehorses [120]. Finally, in the first postmortem study mentioned above, the erosion of enamel and dentine of the first mandibular premolar, indicative of bit wear, was observed in 62% of 29 working horses [119]. Likewise, in the third postmortem study above, premolar erosion was observed in 61% of the 66 domestic horse mandibles, such that, overall, 88% of those mandibles exhibited either bone spurs or premolar erosion, or both [7].

The formation of bone spurs in affected horses is apparently due to inflammation associated with repeated incidents of bit-induced bruising, laceration, and/or ulceration of the interdental gums [7,121,122,123]. Such gum lesions reportedly occurred in 26% of 261 Trotters observed after a race [124], increased from 8% before to 31% after events in 77 competition horses [125], and were more common and severe in racehorses than polo ponies [120]. All such lesions are painful [107], and human experience would suggest that the intensity of that pain would be increased when there is further direct compressive contact between these lesions and a bit. Likewise, in view of the dense nociceptive innervation of the dentine and, to a lesser extent, the tooth pulp [126], further compressive bit contact with significantly worn teeth (mentioned above [7,119]) would also be likely to increase the intensity of any associated pain.

#### 4.1.2. The Tongue

The tongue, being densely supplied with mechanoreceptors [99], exhibits exceptional tactile sensitivity which underlies its haptic functions of delicate investigation and selective manipulation of food and other objects both inside and outside the mouth. It is also well supplied with nociceptors, although a reported low responsiveness of horses to severe lacerations in the mobile rostral portion of the tongue, amounting in some cases to near amputation [104,127], suggests that nociceptor density in the tongue may be less than in the periosteal gums of the interdental space [99,107,128]. However, this does not imply that the tongue is insensitive to painful stimuli, because injuries such as puncture wounds, abscesses, or ulcers located caudally in the tongue can apparently cause enough pain to seriously impede chewing and swallowing [127].

Nevertheless, several observations suggest that the tongue may be somewhat protected from bit-induced penetrative injuries. Studies that reveal significant bit-related injuries at multiple oral sites report no or very low occurrences of significant tongue lacerations or ulcers [120,124,125,129,130]. The tough keratinized squamous epithelial lining of at least the dorsal surface of the tongue [131] may contribute to this, but it might also make bruises from non-penetrative bit-related compression more difficult to detect. 

Bruising of the tongue would likely occur at its lateral edges when the horse uses it to partially cushion the interdental gums against significant bit pressure. Under bit pressure, the tongue may lie ventrally across the full width of the oral cavity covering the interdental gums on each side, such that, at its edges, the tongue may become painfully compressed between the bit and the mandible [11,125,132]. Although this might reduce the overall pain experienced, it would not eliminate it. This is because narrow under-the-bit compression across the width of the tongue between its lateral edges would still be painful, and some pain-inducing nociceptor stimulation may still occur within the highly pain-sensitive interdental spaces (see Section 4.1.1), despite cushioning by the tongue.

Another strategy apparently deployed by horses to ameliorate bit-induced pain is to manoeuvre the tongue to lie above or behind the bit [11,133,134]. The position above the bit would potentially enable the frenulum and adjacent sublingual tissues to absorb some of the bit pressure generated by rein tension. Although this would itself be painful, this strategy may be sufficient to reduce the bit pressure applied directly to the interdental gums and/or to the premolars for the outcome to be a net reduction in pain. That a significant proportion of horses utilize this “tongue over the bit” strategy is indicated by the relatively frequent use of tongue ties to prevent them from doing so [134,135,136]. Thus, 72% of Thoroughbred trainers in Australia reportedly used tongue ties with over 30% of horses wearing a tongue tie at least once [134]. Moreover, once applied to a racehorse, a tongue tie was used in 84% of their subsequent races. Overall tongue tie use was greater in jumps races (45%) than in flat races (32%) [136]. In the United Kingdom, tongue ties were used over a 2-year period in 5% of horses, and after being used once they were applied in an average of 77% of the races run by those horses during the following year [135]. Advocates for this intervention often proffer the justification that “tongue-tied” racehorses are more responsive to the bit and are therefore easier to control, and/or that they are less susceptible to compromised breathing resulting from dorsal displacement of the soft palate which impedes their racing performance [134,135,136,137,138]. Note however that contrary evidence exists, which shows that bit-induced mouth pain makes many horses difficult to control [11] (Section 5).

Nevertheless, regarding the greater purported sensitivity to the bit and effectiveness of control, advocates of this intervention reason that: (1) bit-induced mouth pain is used to control potentially unruly horses; (2) some horses relocate their tongues over the bit to alleviate the pain; (3) “tongue over the bit” horses are less responsive to the bit and are therefore harder to control; and (4) when tongue ties are used to restore a “bit over the tongue” configuration, responsiveness to the bit and effectiveness of control return. However, so does a greater intensity of bit-induced pain. It therefore follows that tongue tie use enables its advocates to impose on horses, or threaten them with, bit-induced pain at noxious intensities designed to achieve the sense of control they seek. Note in addition that use of tongue ties is itself aversive and likely adds significantly to the pain.

Tongue ties are usually applied by grasping the tongue, drawing it sideways out of the mouth, winding the tie around the tongue one or more times and securing it below the mandible ventral to the interdental space; the purposes are to hold the tongue flat against the ventral surface of the oral cavity and to stop it from being retracted [139]. Nylon stocking, leather, or rubber bands are used. It is common for a length of tongue beyond the tie to protrude from the horse’s mouth. Problems with tongue tie use, reported by nearly a quarter of Australian Standardbred trainers, include lacerations, bruising and swelling of the tongue, difficulty swallowing, and stress behaviours [139,140]. It is proposed here that the stress behaviours indicate pain-related aversion to the tie. The likely sources of significant pain include the following: lengthwise over-stretching of the tongue during application of the tie; compression of the tongue directly under the tie; impeded blood flow to the rostral tongue while the tie is in place and its restoration when the tie is removed (ischaemic pain); and pain linked to any bruising and lacerations. In addition, the tie narrows the tongue medially, which prevents it from overlying the interdental space on each side, thereby increasing the likelihood that, under rein tension, the bit would have direct contact with the highly pain-sensitive periosteal gums (see Section 4.1.1). 

#### 4.1.3. The Commissures of the Lips and the Buccal Mucosa

The incidence of commissure lesions has been reported in several studies. (1) Acute lesions were apparent in 64% of Finnish trotters, where blood was visible on the bit or the wound in 10% that had the most severe lesions [124]. (2) About 9% of Danish horses in dressage, show jumping, eventing, and endurance competitions had commissure lesions, some of which were accompanied by visible blood [130]. (3) In 50 polo ponies and 50 racehorses, commissure ulcerations numbered 15 and 53, respectively, where both the prevalence and severity of the lesions were greater in the racehorses than the polo ponies [120]. The prevalence in these racehorses was later estimated to be ~25% [124]. (4) Mild, apparently older lesions, both inside the commissures and in the adjacent buccal mucosa, were found in 26% of Icelandic horses prior to prescribed gait competitions, and a further 4% had severe lesions in the buccal mucosa [125]. And (5), buccal ulceration or evidence of previous ulceration adjacent to maxillary molars was apparent in 94% of ridden Swedish horses [129]. These observations and the finding that no fresh lesions were observed in brood mares that had not recently been used wearing a bitted bridle [129] implicate bit use as a cause [124].

Commissure and adjacent buccal bruising, laceration, ulceration, and bleeding provide clear evidence of prior damaging impacts of bit pressure on the nociceptors of the internal mucosal and external lip tissues sufficient to cause significant pain. Moreover, this pain would be intensified by bit and/or molar tooth pressure on any recently formed lesions. A further indication of high bit pressure on the commissures is the readily observable stretching of the lips to up to double their resting non-bitted length when rein tension is applied [1,11,96,97,141]. Note that this stretching, whether short-lived or sustained, would itself cause pain. The reader may confirm this by repeating the “Mellor pen test” position 2 (Figure 1B), but instead of applying little pressure, for this purpose the pen should be pushed carefully towards the back of the mouth as far and for as long as the induced lip-stretching pain will allow.

### 4.2. General Comments and Summing up

The reported post-race prevalence of lesions over the full range of severity at all oral sites was 84% in Finnish trotters [124], 88% in Swedish trotters [129], and in three studies of Icelandic horses after competition events it was 60% in 2012, 33% in 2014, and 43% in 2016 (for references see [124,125]). To date, with few exceptions (e.g., [124]), key publications that have dealt specifically with bit-induced oral trauma either did not mention pain at all or made only fleeting reference to it (e.g., [7,108,109,110,111,112,113,114,115,116,117,118,119,120,125,129,130]. Nevertheless, it is apparent from the preceding analysis that the principal welfare issue here is pain.

All oral sites referred to above are richly supplied with nociceptors and are susceptible to bruising, laceration and ulceration. The prevalence of these lesions clearly indicates that, notwithstanding many riders’ specific intentions to the contrary, rein tensions transmitted to the bit may often cause tissue trauma and associated pain at intensities that are of welfare concern. Note, in addition, that the periosteal gums of the interdental space are especially sensitive to noxious stimulation. In fact, they are so sensitive that low bit pressures which would not produce detectable lesions can still cause significant pain, as indicated by the “Mellor pen test” (Figure 1), and higher pressures that do produce visible lesions would cause marked to extremely severe pain.

It is noteworthy that once lesions at any oral site have developed, repeated direct contact with the bit would magnify the intensity of the resulting pain [22,142], whether the bit pressure is transient or sustained, is applied abruptly or slowly, or repeatedly oscillates up and down during the rhythmic step phases of the trot or canter [110,111,112,113,114,115]. Moreover, inflammatory reactions in and around the lesions would likely lead to the development of pain hypersensitivity due to decreases in nociceptor stimulus thresholds within the lesions and nearby tissues (see [22,142] for details of how pain experience changes after injury). Moreover, the persistent aggravation of lesions and the nearby inflamed tissues by repeated bit contact would delay healing and resolution of any associated pain (see [124] for references]). Finally, protracted, repetitive, and noxious oral stimulation may lead to a more widespread and lasting hypersensitivity in the form of trigeminal neuralgia, which, recognised behaviourally, manifests as recurring episodes of sudden, sharp, and exceptionally intense pain experienced in various facial locations remote from the mouth [1,5,11,143], episodes which may be triggered at both oral and non-oral locations [144,145]. 

It is beyond the scope of this review to consider the impact of bit design on these phenomena. Suffice it to say here that oral contact sites and thus the location and severity of lesions appear to depend on particular design features of different bits and how the bits are used [104,106,120,124,125,129,130,133]. However, with some exceptions (e.g., [106,133]), many investigations are handicapped by having to rely on horses that have been made available by owners who supply them wearing their own tack when participating in various independently scheduled equestrian activities, so that rigorous comparisons of the specific impacts of different bit types can be difficult. 

## 5. Behavioural Indices of Mouth Pain in Horses

As noted above (Section 2.5), behaviour is often used to indicate when animals, including horses, are in pain. Some behavioural responses to mouth pain may be identified easily as being due to noxious oral stimuli, whereas the link with other responses may not be as obvious. This is because indicative behaviours may involve the mouth, tongue, lips, nostrils, eyes, ears, head, neck, trunk, legs, and/or tail, as well as changes in posture, gait, and the vigour and character of locomotory activity. The available information for the present analysis, summarised in Table 2, has been presented with three overlapping orientations: first, behaviours of bitted horses, especially those involved in competitive athletic events; second, behavioural changes when horses are transitioned from being bitted to bit-free; and third, bit-free behaviour, in particular that of domesticated horses wearing halters or no tack, and that of wild, free-roaming horses. [Table 2 near here]

On the basis of detailed behavioural observations (Table 2), a bit in a horse’s mouth at zero rein tension might appear to be accepted by the horse or may merely be tolerated as a mild irritant. However, as rein tensions rise, the bit clearly becomes increasingly aversive because the horse is confronted with escalating inescapable pain (Section 4). Abrupt, highly aversive increases in rein tension often occur when a sharp change of direction or speed is elicited, for example, during competitive events requiring agility such as barrel racing, calf roping, and polo matches [10]. Though somewhat less abrupt, frequent changes in rein tension commonly occur during competitive cross-country and show jumping events [10]. In contrast, elevated rein tensions are often sustained for at least the first half of flat races, steeplechase, and harness races until the horses are “given their heads” to accelerate towards the finish line, after which they are again “reined in” when jockeys seek to reduce their speed to a walk [10]. Some pain-induced behaviours may also be apparent during events that primarily focus on deportment and demeanour at low speed, in particular dressage and some draft horse competitions [10]. However, it is not suggested here that throughout every ride horses would continuously experience significant pain, but it is clear that under the circumstances just described highly aversive levels of pain would be experienced with the rein tensions known to be used.

It is recommended that readers assess the behavioural evidence outlined in Table 2 for themselves and draw their own conclusions. YouTube videos in particular are a rich resource [10]. Filmed independently, they provide objectively observable records of equine behaviour in all of the circumstances referred to above, and many more. Likewise, equine events are regularly screened on television. Finally, whether they participate as equestrians or not, readers who personally attend these events or who are recreationally involved with less formal equine activities may make their own direct observations of the behaviour of horses wearing bitted and bit-free bridles, halters, or no bridles at all. 

It should be noted that the bit-free bridles referred to here are those that are loosely and comfortably fitted and are used in ways that are intended to be pain-free (e.g., [11,148,161,162]). At their best, therefore, they do not replace the control of horses via bit-induced mouth pain with control via rein tension conveyed to rigid or tight bridle straps in contact with sensitive parts of the face or head, such as the muzzle, nose, jaw, and/or poll [4,163,164]. Accordingly, their use contrasts sharply with the consequences of firm-handed rein pressure on the bosal-like nosebands of hackamore bridles [165,166], or on other bit-free bridles designed with tightly fitting or rigid nosebands or straps [161,163,164].

Those readers who engage in an exploration of the pain-related behaviours noted in Table 2 will quickly discover that most horses do not display all of them at once, or over an extended period. For example, among the 69 such behaviours identified by the riders of 66 horses that were changed from bitted bridles to a bit-free bridle, before the change only 57 exhibited the most prevalent combination of behaviours described as “hates the bit”, 43 were “not controllable”, 37 engaged in “head shaking”, 33 were “difficult to steer”, 32 engaged in “choppy striding”, 31 in “tail swishing’, 29 in “hair trigger responses”, 25 had their “mouth gaping open”, 24 had “anxious eyes”, 23 “grabbed the bit”, 20 “bucked”, and 12 had their “tongue over the bit” [11]. Nevertheless, 65 of the 66 horses exhibited aversion to the bit in a total of 69 ways, which were considered to express their immediate responses to the bit-related pain and/or their frustration at thwarted attempts to avoid it [11]. In contrast, and importantly, these behaviours and others referred to in Table 2 were absent or rarely observed in ridden horses transitioned from wearing bitted to bit-free bridles, and in domesticated or free-roaming wild horses wearing no tack.

It is widely acknowledged among equestrians that some horses show just a few signs of aversion to the bit; what is not acknowledged is that every horse has the potential to be averse to the bit as a foreign body in its mouth and that horses have many ways of expressing that aversion [11]. In part, this lack of acknowledgement is due to what the present author calls “bit blindness”. This is a descriptive term, not a critical or pejorative one. Its purpose is to highlight a widespread lack of recognition that the distinctive behaviours described here (Table 2), which are observable almost every day, are in fact specific indices of bit-induced mouth pain. Note however that such “bit blindness” really reflects a misinterpretation. It arises because bit use and the associated behaviours have been part of human–horse interactions for at least four millennia [167]. Thus, it is suggested here that a pervasive familiarity has led to a perception that these regularly observed behaviours are natural to the horse, being little to do with the presence of a bit. The persistence of this perception down the years has quite understandably influenced the vast majority of equestrians who are active today. A similar phenomenon has been observed with dairy cattle. Apart from the most severe cases, dairy farmers markedly underestimated the proportion of lame cows in their herds. After being shown the behavioural signs of less severe lameness, many of them said, “I thought cows just walked that way” [168,169,170]. Once fully recognised, however, the signs of bit-induced mouth pain in horses, as with lameness in dairy cows, cannot be “unseen”. Nevertheless, resolute defenders of the previously prevalent view might even then use minimising, distracting, or euphemistic words or phrases to divert attention from what these behaviours actually indicate [170]. When these behaviours are considered in the context of the whole analysis conducted here, their meaning is clear—equestrians whose approach is to firmly control horses using bitted bridles will often, even if unintentionally, cause them pain, sometimes very severe pain.

## 6. Welfare Implications of Bit-Induced Mouth Pain in Horses

The evidence-based analysis conducted here shows unequivocally that bit-induced mouth pain is likely to be a significant cause of welfare compromise in the majority of conventionally bridled horses. Moreover, the greater the rein tension, whether abruptly applied, short-lived, sustained, or cyclical, the greater will be the following factors: the noxiousness of the immediate pain experience; the likelihood of tissue trauma and the associated continuing pain; the intensity of any pain elicited by later bit contact with the tissues injured previously; and the time required for those lesioned tissues to heal. Nevertheless, as already stated, it is not suggested here that throughout every ride horses would continuously experience significant pain, but it is clear that under most of the competitive circumstances described above (Section 5), highly aversive levels of pain would be experienced with the stronger rein tensions known to be used.

Yet, there are even wider welfare consequences than the direct impacts of the pain experience itself. They relate to specific behaviours elicited by the bit-induced pain and involve the following factors: the horse’s open mouth; its tongue relocated over the bit or retracted behind it; and when initiated by the rider or driver, the presence of low jowl angles maintained by firm application of rein tension. In animal welfare terms, they all lead to compromised breathing and unpleasant, sometimes exceptionally unpleasant sensations of breathlessness, experienced by people as suffocation [171]. The reader is referred to the previous full account of these phenomena [10] in order to access the 164 published sources that underlie the following brief explanation.

### 6.1. Respiratory Consequences of an Open Mouth and Relocation of the Tongue above or behind the Bit

**Figure 2:**
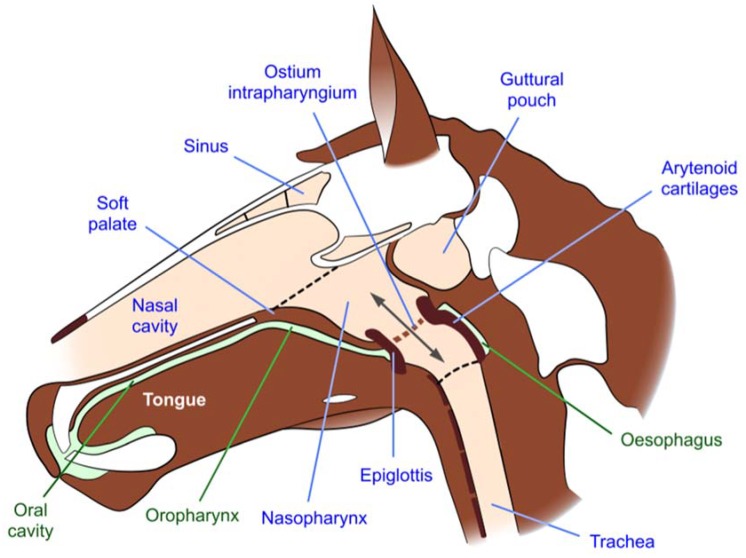
Diagram of the relationship of the soft palate and the larynx of the horse while breathing with its mouth closed (modified from [146] with permission). The larynx (the “button”) fits tightly into the ostium intrapharyngium (the “buttonhole”) of the soft palate, creating an airtight seal so that air cannot enter the oropharynx. This, and closed lips, enables a negative pressure to be maintained in the oral cavity and oropharynx, which holds the soft palate against the root of the tongue, thereby widening the nasopharyngeal airway. Disengagement of the soft palate and larynx and/or loss of the lip seal dissipate the negative pressure in the oral cavity and oropharynx, which then allows the soft palate to rise, vibrate with each breath, and impede nasopharyngeal airflow. The double-headed arrow indicates the directions of airflow. Reproduced from [10], also published by *Animals*.

Unlike people, dogs, ruminants, and most other mammals, horses are “obligate nasal breathers”. For fully effective respiration they must breathe through their noses and, being exceptional athletes, the physiological demands on their respiratory systems are substantial. For example, in order to meet the oxygen demands of vigorous muscular activity when at full gallop, Thoroughbred racehorses must breathe in and out 110–130 times a minute, achieving total airflows of 1800–2000 L/minute, which represent a 25–27 fold increase on the values at rest. This is equivalent to breathing in and out 180–200 10 L buckets of air every minute. To achieve this, the respiratory passages need to be as widely open as possible, as even minor obstructions disproportionately impede airflow in accord with Poiseuille’s Law [172]. This is largely achieved by the creation of negative pressure in the oral cavity and oropharynx by swallowing with the mouth closed, and keeping it closed [146,173,174]. This negative pressure holds the soft palate firmly down onto the root of the tongue deep in the throat (Figure 2), and requires the establishment of airtight seals at the lips with the mouth closed and, deep in the throat, with the larynx fitting tightly into the soft palate orifice (the ostium intrapharyngium) (see the legend of Figure 2 for a more detailed explanation). If one or both of these seals is broken, air enters the oral cavity and oropharynx, freeing the soft palate to balloon up into the nasopharynx, where it vibrates at each breath, impeding airflow. A bit-induced mouth opening, even a small opening, breaks the lip seal, and the bit-induced bulging of the tongue deep in the throat can also break the palato-laryngeal seal [146,173,174]. Palatal instability results, and this may progress in steps of increasing severity to an extreme of palato–laryngeal disengagement in which the soft palate is drawn above the epiglottis, partially or completely blocking airflow during inspiration and impeding it on expiration [146,173,174]. Clinically described as dorsal displacement of the soft palate (DDSP), this upper airway impediment to airflow initiates a cascade of pathophysiological changes in the lower airways [10]. Recognised as exercise induces pulmonary haemorrhage (EIPH), proposed to be one feature of negative pressure pulmonary oedema (NPPO) [10,173], these changes include increased airflow resistance in the lower airways and/or impeded respiratory gas exchange in the alveoli [175,176,177]. It is these effects that generate the subjectively unpleasant, and therefore welfare-compromising experiences of breathlessness, which human patients with NPPO describe as intense feelings of suffocation [171].

### 6.2. Respiratory Impacts of Low Jowl Angles Maintained by the Firm Application of Rein Tension

The jowl angle is the angle of intersection of the leading edge of the neck and the line of the lower jaw. The jowl angle of a horse at rest and unconstrained by rein tension would normally be about 90° or slightly more. When galloping, it may cyclically extend its head-neck to jowl angles that approach 120° [146]. This straightens and widens the nasopharynx and disproportionately reduces nasopharyngeal airflow resistance (Poiseuille’s Law: [172]); it also stretches and straightens the extrathoracic trachea, which makes it less susceptible to dynamic collapse during inspiration. On the other hand, jowl angles of less than 90° are accompanied by reduced cross-sectional areas of the nasopharynx, which disproportionately, and markedly, increases airflow resistance and decreases airflow rates [146,172], as well as alveolar gas exchange [10]. The extent of compromised breathing at jowl angles of ~33° when the nasal plane is nearly vertical, or of < 33° when the nasal plane is behind the vertical as in the Rolkur position [8], is likely to generate intense feelings of breathlessness.

It is apparent that the unnaturally low jowl angles seen during dressage, and the extreme of Rolkur, are achieved by high rein tensions causing significant mouth pain [115,178,179]. This is also likely with the low jowl angles often observed, albeit transiently, during different phases of show jumping and other events (Table 2 and YouTube videos). However, the threat of a return of marked bit-related pain experienced during early dressage and other training may motivate the horse to cooperatively adopt these lower jowl angles in response to lower rein tensions than were originally required. The following observation is consistent with this suggestion. Dressage riders maintained higher mean bit pressures of ~6.6 kg/cm^2^ by continuously applying rein tension, whereas, when the required jowl angles were maintained by reins of constant length secured to a surcingle frame, the horses self-selected lower mean bit pressures of ~2.1 kg/cm^2^ by marginally reducing their jowl angles themselves (bit pressures were calculated from reported rein tension data of [115]). Nevertheless, although the lower self-selected bit pressures would also have been painful, albeit less so, the low jowl angles would still have compromised airflow and likely generated unpleasant experiences of breathlessness [10,171].

### 6.3. Pain-Related Conflict Behaviours and Summing up

Conflict behaviours are characterised as a response of horses that are apparently having difficulty coping with mental or physical discomfort, reflected in some form of resistance to handling or training cues and/or to equipment [180]. All such behaviours are absent in wild, free-roaming horses [66,160], but are characteristic of the ridden horses (Table 2). Typical examples of these conflict behaviours include head shaking, mouth gaping open or resisting bit contact, tugging or pulling the reins out of rider’s hands, and excessive tail swishing during ridden activities [115,152,153,154,157]. It is apparent that these behaviours are the same as some of those elicited by bit-induced mouth pain (Table 2), which suggests that mouth pain may be at least one of several factors that underlie conflict behaviours. It is suggested here that anxiety in anticipation of pain, and fear whilst experiencing it, especially if the pain is intense, may be additional emotional constituents of conflict behaviours. Moreover, anxiety may also accompany the experience of suffocating breathlessness in circumstances when it is anticipated, and fear when it actually occurs [30,171].

The above observations support the conclusion that, in addition to the direct impacts of bit-induced mouth pain, the associated negative subjective experiences of breathlessness, anxiety and fear are also likely to be components of the associated animal welfare compromise [10,70].

## 7. A Suggested Solution

The foregoing analysis shows that there is no longer any need to ask, “Do we have a ‘bit’ of a problem?” [118], because we clearly do. Moreover, the analysis provides a compelling case for taking decisive steps to seek real solutions. 

What the present author describes as “straitjacket solutions” will not be of assistance. Although they diminish some of the problematic behaviours they are specifically aimed at controlling (see [179] for further critique), they do not reduce the pain which is the root cause. Examples include the following: the use of martingales for head tossing; bits with flanges to minimise relocation of the tongue over the bit or its bulging upwards in the throat; tongue ties to keep the tongue forward and flat on the ventral surface of the mouth; and exceptionally tight nosebands to keep the mouth closed. Note that at least two of these interventions may generate extra pain; tongue ties by stimulating lingual nociceptors (Section 4.1.2) and exceptionally tight nosebands by stimulating skin-periosteal nociceptors at pressure points on the nose and jaw (e.g., [8,179,181,182,183]).

A remedy does appear to be available by discontinuing the use of the bit and utilising bit-free bridles. The Bedouin of North Africa and some American Indians commonly rode bit-free [148,164]. During the last two-to-three decades, bit-free riding of sport and recreational horses has burgeoned, and this has been accompanied by a progressive increase in scientific comparisons of bitted and bit-free riding (see Table 2). New designs of bit-free bridles, the use of which is specifically intended to be pain-free, continue to be developed (e.g., [162]). Accordingly, there is a considerable amount of experience and information available that could guide the wider use of such bridles. 

Those unfamiliar with bit-free riding or driving often raise concerns that without a bit, the horse being a strong and potentially unruly animal, would be uncontrollable and a danger to itself and to riders, drivers, and bystanders. This is the dominant view that underlies the current requirement for Thoroughbreds and harness horses to be controlled by a bit while racing. This is also reflected in the wider conviction that without a bit horses participating in numerous other competitive events could not be controlled safely, nor with the agility and/or precision desired in each case.

So, how might we proceed? We cannot simply ignore the bit problem, which has now been identified so clearly. Inaction when a problem is not apparent is understandable. Inaction once a significant problem has been recognised is unacceptable. Recognition of such a problem brings with it an ethical responsibility to act.

Outlined below are 12 questions designed to guide specific steps that could progress this matter. Brief comment is made on each one based on more than 20 years of experience of numerous individuals who have engaged in bit-free recreational and other riding.

1. What specific competitive events have been tested for control and safety using bit-free bridles?
  It appears that no data for comparing the responses of horses ridden bitted or bit-free have been collected, although opportunities for doing so are available with dressage. Bit-free dressage, excluding Grand Prix, is supported in the Netherlands [184], and two online American Western dressage associations give riders the choice of competing using bitted or bit-free bridles [185,186]. In addition, it would be worthwhile to set up other studies that compare the safety and control of horses ridden bitted and bit-free in other competitive events, including, but not limited to, flatraces, steeplechases, and harness races. However, the requirement that bits must be worn during such racing events means that off-track trials would probably be needed.
2. Have horses trained from the outset to be ridden bit-free been tested in competition for their ease of control and whether or not they can be ridden safely?
  Not yet, but sufficient time has passed since bit-free riding became more popular for there to be many young horses that would be eligible for such testing.
3. Can horses trained from the outset to be ridden with bitted bridles be successfully transitioned to being ridden safely bit-free?
  Yes, many years of successful transitioning of horses for recreational riding attest to this, as do a number of published studies (Table 2). However, additional studies would be beneficial.
4. If so, what proportion of horses are not able to be transitioned to bit-free riding?
  Evidence is lacking on the proportion of horses that cannot be so transitioned, but anecdotal reports from horse owners or riders familiar with bitted bridle use suggest that a large majority of horses do respond well when ridden bit-free [11]
5. In the horses that can be transitioned, how long does it take?
  Owners or riders report that benefits are apparent during the first day and that further improvements occur during subsequent days or weeks [11,147].
6. Are there particular bit-free bridles that improve the success rate of making this transition?
  No data are yet available. It would be anticipated that there would be greater benefits with bit-free bridles that do not cause pain.
7. What are the detailed specifications of the most effective bit-free bridles?
  Design provisions should not be aimed specifically at causing pain, nor should equipment permit pain to be inflicted, even when misused.
8. Are there particular trainers who are more successful with transitioning horses to bit-free riding?
  This would almost certainly be the case, but none have yet been compared so none have been documented.
9. If so, what training of trainers is necessary to increase the number who are successful?
  The content of training programmes would at least partly depend on the outcomes of the foregoing trials. Presumably such training would involve adequate study of available literature combined with knowledgeable hands-on supervision and involvement. Equestrian organisations could consider sponsoring and encouraging the conduct of training workshops and other such activities in teaching establishments.
10. Can riders be trained so that they can successfully transition their own horses to bit-free riding?
  The answer is yes, but only if they are well informed, willing to take advice, and are well motivated.
11. What proportion of horses can never successfully make this transition?
  As yet, there are apparently no anecdotal or other reports of complete failure to make the transition. The foregoing trials would be expected to provide specific information about success or failure rates, and possible reasons for any failures.
12. What is the relative performance of horses ridden bitted and bit-free in different competitive events?
  Although not yet determined rigorously, anecdotal reports suggest that improvements in performance can be expected. This needs to be investigated in carefully designed studies in which the athletic performance of bitted and bit-free horses is compared during time-based competitions, such as in the cross-country and show jumping elements of three-day-events. This also applies to flatraces, steeplechases, and harness races, but, as noted above, the requirement that bits be worn in such events will probably mean that off-track trials will need to be arranged.

Each of these questions is equally applicable to the bit-free driving of harnessed horses, including competition Trotters and cart horses. 

Regarding the last of these questions, the drive to make this transition would likely be boosted greatly if horses ridden or driven bit-free were shown to athletically outperform those wearing bitted bridles. However, this should not be made a precondition for addressing the previous 11 questions. The compelling case made here for actively taking remedial action regarding bit-induced mouth pain in horses demands that all of these questions be addressed rigorously and with scientifically robust study designs.

## Figures and Tables

**Table 1:** Major attributes of pain (modified from [12]).

Attribute	Description
Purpose	Pain is understood to have evolutionary survival value.
Detection	Pain sensations depend on activation of a discrete set of receptors (nociceptors) by noxious stimuli.
Perception	Further processing via nerve pathways to the brain and within the brain enables the noxious stimuli to be consciously perceived as pain.
Character	Pain perception varies according to site, duration and intensity of stimulation and can be modified by previous experience, emotional state and innate individual differences.
Definition	Pain is defined as an unpleasant sensory and emotional experience associated with actual or potential tissue damage, or is describable in terms of such damage.
Variation	The pain-detection threshold is fairly uniform in mammals, whereas pain tolerance may be more species-specific and subject to modification.

**Table 2:** Some behavioural indices of bit-related mouth pain in horses.

**Indicative Pain-Related Behaviours in Ridden Bitted Horses**
*Mouth:* resists bridling; fussing with the bit, persistent jaw movements, chewing; crossing the jaw; slightly open or gaping mouth; teeth grinding, holding the bit between the teeth; tongue persistently moving or protruding from the mouth, tongue placed above the bit or retracted behind it; excessive salivation or drooling. *Head-neck:* sudden evasive movements due to abrupt increases in rein tension; side-to-side or up-down head shaking, jawline above horizontal; head tilted, stiff necked; rein-induced low jowl-angle, neck arched, nasal plane at or behind the vertical; reaches forward so rider uses longer rein. *Pain face:* identifiable nostril flare, lip positions, ear positions, eye white visibility and facial muscle tension. *Body movement/gait:* stiff or choppy stride, hair trigger responses, crabbing; difficult to control, hesitant to move forward, difficult to stop, side-stepping from straight-line motion; bucking; rearing; tail swishing. Refs: [6,28,83,90,96,97,115,133,140,141,146,147,148,149,150,151,152,153,154,155,156,157,158]; plus YouTube archive videos ^a^
**Bitted to Bit-Free Changes in Ridden Horse Behaviour**
*Mouth:* all bit-related mouth behaviours absent; quiet, closed mouth, tongue inside mouth and appropriately placed; little or no teeth grinding; no drooling. *Head-neck:* head shaking absent; lower head-neck position and wider jowl angle; head, neck and spinal column properly aligned longitudinally. *Pain face:* no indications of mouth-related pain in healthy animals. *Body movement/gait:* calm, relaxed and cooperative demeanour; engaged, lively, energised and exhibits vitality of fitness; head freedom supports balanced, aligned and smooth rhythm of motion; tail movement in synchrony with spinal movement. Refs: [1,4,9,11,147,148,149,150,151]; plus YouTube archive videos ^a^
**Behaviours of Bit-Free Horses at Rest or When Running Free**
As expected, domesticated horses wearing loosely-but-snugly fitted bit-free bridles do not display any of the bit-related behaviours noted above while standing at rest or engaging in exercise ranging from walking to galloping; nor do horses wearing halters while standing in stalls or moving freely in turnout paddocks. Likewise, neither do wild, free-roaming horses when standing alert or when walking, trotting, cantering and galloping during roundups. Refs: [159,160]; YouTube archive videos of bit-free domesticated horses, and of ~150 free-roaming, wild Brumbies (Australia), Camargue horses (France), Kaimanawa horses (New Zealand) and Mustangs (USA) ^a^

^a^ Google “YouTube plus the named activity or event for competition horses”, or “stipulate documentaries and roundups about bit-free, wild or free-roaming horses”, then follow links to the numerous filmed records [10].

## References

[1] Cook W.R. (2003). Bit-induced pain: A cause of fear, flight, and facial neuralgia in the horse. Pferdeheilkunde.

[2] Cook W.R., Strasser H., Kells S. (2003). Harmful effects of the bit. Metal in the Mouth: The Abusive Effects of Bitted Bridles.

[3] McLean A.N., McGreevy P.D., Mills D.S., McDonnell S.M. (2005). Behavioral problems with the ridden horse. The Domestic Horse: The Origins, Development, and Management of Its Behaviour.

[4] Quick J.S., Warren-Smith A.K. (2009). Preliminary investigation of horses’ (*Equus caballus*) responses to different bridles during foundation training. J. Vet. Behav..

[5] Hannah C. The Truth about Bits: Facial [Trigeminal] Neuralgia. *Horse and Human* 2009. http://www.horseandhuman.co.nz/articles/html/the_truth_about_bits_part4.html.

[6] Williams L.R., Warren-Smith A.K. (2010). Conflict responses exhibited by dressage horses during competition. J. Vet. Behav. Clin. Appl. Res..

[7] Cook W.R. (2011). Damage by the bit to the equine interdental space and second lower premolar. Equine Vet. Educ..

[8] McGreevy P.D. (2011). The fine line between pressure and pain: Ask the horse. Vet. J..

[9] Jahiel J. Increase Comfort, Reduce Risk: The Bitless Bridle. *Equestrian Medical Safety Association* 2014, Fall Newsletter. http://emsaonline.net/wp-content/uploads/gravity_forms/1-5f7def.

[10] Mellor D.J., Beausoleil N.J. (2017). Equine welfare during exercise: An evaluation of breathing, breathlessness and bridles. Animals.

[11] Cook W.R., Kibler M. (2019). Behavioural assessment of pain in 66 horses, with and without a bit. Equine Vet. Educ..

[12] Merskey H. (1979). Pain terms: A list of definitions and notes on usage. Pain.

[13] Mellor D.J., Cook C.J., Stafford K.J., Moberg G.P., Mench J.A. (2000). Chapter 9: Quantifying some responses to pain as a stressor. The Biology of Animal Stress: Basic Principles and Implications for Welfare.

[14] Sneddon L.U., Elwood R.W., Adamo S.A., Leach M.C. (2014). Defining and assessing animal pain. Anim. Behav..

[15] McLennan K.M., Miller A.L., Dalla Costa E., Stucke D., Corke M.J., Broom D.M., Leach M.C. (2019). Conceptual and methodological issues relating to pain assessment in mammals: The development and utilisation of pain facial expression scales. Appl. Anim. Behav. Sci..

[16] Molony V. (1997). Comments on anand and craig, pain 67 (1996). Pain.

[17] Mellor D.J., Stafford K.J. (2000). Acute castration and/or tailing distress and its alleviation in lambs. N. Z. Vet. J..

[18] Stafford K.J., Mellor D.J. (2005). Dehorning and disbudding distress and its alleviation in calves. Vet. J..

[19] Stafford K.J., Mellor D.J. (2005). The welfare significance of the castration of cattle: A review. N. Z. Vet. J..

[20] Weary D.M., Niel L., Flower F.C., Fraser D. (2006). Identifying and preventing pain in animals. Appl. Anim. Behav. Sci..

[21] Prunier A., Mounier L., Le Neindre P., Leterrier C., Mormede P., Paulmier V., Prunet P., Terlouw C., Guatteo R. (2013). Identifying and monitoring pain in farm animals: A review. Animal.

[22] Gregory N.G. (2004). Physiology and Behaviour of Animal Suffering.

[23] Wynne C.D.L. (2007). What are animals? Why anthropomorphism is still not a scientific approach to behavior. Comp. Cogn. Behav. Rev..

[24] Serpell J.A. (2019). How happy is your pet? The problem of subjectivity in the assessment of companion animal welfare. Anim. Welf..

[25] Bermond D.G. (2001). A neuropsychological and evolutionary approach to animal consciousness and animal suffering. Anim. Welf..

[26] Key B. (2016). Why fish do not feel pain. Anim. Sentience.

[27] Dawkins M.S. (2017). Animal welfare with and without consciousness. J. Zool..

[28] Waran N., Randle H. (2017). What we can measure, we can manage: The importance of using robust welfare indicators in equitation science. Appl. Anim. Behav. Sci..

[29] Lesimple C. (2020). Indicators of horse welfare: State-of-the-art. Animals.

[30] Mellor D.J. (2019). Welfare-aligned sentience: Enhanced capacities to experience, inte ract, anticipate, choose and survive. Animals.

[31] (2019). Critical Anthropomorphism. *Wikipedia*. https://en.wikipedia.org/wiki/Critical_anthropomorphism.

[32] Talbot J.D., Marrett S., Evans A.C., Meyer E., Bushnell M.C., Duncan G.H. (1991). Multiple representations of pain in human cerebral cortex. Science.

[33] Jones A.K.P., Friston K., Frackowiak R.S.J. (1992). Localization of responses to pain in human cerebral cortex. Science.

[34] Treede R.D., Kenshalo D.R., Gracely R.H., Jones A.K.P. (1999). The cortical representation of pain. Pain.

[35] Adrian E.D., Matthews B.H.C. (1934). The interpretation of potential waves in the cortex. J. Physiol..

[36] Coole J.W., Tukey J.W. (1965). An algorithm for the machine calculation of complex fourier series. Math. Comput..

[37] Chen A.C.N., Dworkin S.F., Haug J., Gehrig J. (1989). Topographic brain measures of human pain and pain responsivity. Pain.

[38] Bromm B., Lorenz J. (1998). Neurophysiological evaluation of pain. Electroencephalogr. Clin. Neurophysiol..

[39] Chang P.F., Arendt-Nielsen L., Graven-Nielsen T., Svensson P., Chen A.C.N. (2001). Topographic effects of tonic cutaneous nociceptive stimulation on human electroencephalograph. Neurosci. Lett..

[40] Chang P.F., Arendt-Nielsen L., Graven-Nielsen T., Svensson P., Chen A.C.N. (2001). Different EEG topographic effects of painful and non-painful intramuscular stimulation in man. Exp. Brain Res..

[41] Chang P.F., Arendt-Nielsen L., Chen A.C.N. (2002). Differential cerebral responses to aversive auditory arousal versus muscle pain: Specific EEG patterns are associated with human pain processing. Exp. Brain Res..

[42] Ong R.M., Morris J.P., O’Dwyer J.K., Barnett J.L., Hemsworth P.H., Clarke I.J. (1997). Behavioural and EEG changes in sheep in response to painful acute electrical stimuli. Aust. Vet. J..

[43] Jongman E.C., Morris J.P., Barnett J.L., Hemsworth P.H. (2000). EEG changes in 4-week- old lambs in response to castration, tail docking and mulesing. Aust. Vet. J..

[44] Murrell J.C., Johnson C.B., White K.L., Taylor P.M., Haberham Z.L., Waterman-Pearson A.E. (2003). Changes in the EEG during castration in horses and ponies anaesthetized with halothane. Vet. Anaesth. Analg..

[45] Murrell J.C., White K.L., Johnson C.B., Taylor P.M., Doherty T.J., Waterman- Pearson A.E. (2005). Investigation of the EEG effects of intravenous lidocaine during halothane anaesthesia in ponies. Vet. Anaesth. Analg..

[46] Haga H.A., Ranheim B. (2005). Castration of piglets: The analgesic effects of intratesticular and intrafunicular lidocaine injection. Vet. Anaesth. Analg..

[47] Johnson C.B., Stafford K.J., Sylvester S.P., Ward R.N., Mitchinson S., Mellor D.J. (2005). Effects of age on the electroencephalographic response to castration in lambs anaesthetised using halothane in oxygen. N. Z. Vet. J..

[48] Johnson C.B., Wilson P.R., Woodbury M.R., Caulkett N.A. (2005). Comparison of analgesic techniques for antler removal in halothane-anaesthetized red deer (Cervus elaphus): Electroencephalographic responses. Vet. Anaesth. Analg..

[49] Johnson C.B., Gibson T.J., Stafford K.J., Mellor D.J. (2012). Pain perception at slaughter. Anim. Welf..

[50] Murrell J.C., Johnson C.B. (2006). 2006 Neurophysiological techniques to assess pain in animals (Review). J. Vet. Pharmacol. Ther..

[51] Gibson T.J., Johnson C.B., Stafford K.J., Mitchinson S.L., Mellor D.J. (2007). Validation of the acute electroencephalographic responses of calves to noxious stimulus with scoop dehorning. N. Z. Vet. J..

[52] Gibson T.J., Johnson C.B., Murrell J.C., Hulls C.M., Mitchinson S.L., Stafford K.J., Johnstone A.C., Mellor D.J. (2009). Electroencephalographic responses of calves to slaughter by ventral neck incision without prior stunning. N. Z. Vet. J..

[53] Gibson T.J., Johnson C.B., Murrell J.C., Chambers P.J., Stafford K.J., Mellor D.J. (2009). Components of EEG responses to slaughter: Effects of cutting neck tissues compared to major blood vessels. N. Z. Vet. J..

[54] Gibson T.J., Johnson C.B., Murrell J.C., Mitchinson S.L., Stafford K.J., Mellor D.J. (2009). Electroencephalographic response to concussive non-penetrating captive bolt stunning in halothane anaesthetised calves. N. Z. Vet. J..

[55] Gibson T.J., Johnson C.B., Murrell J.C., Mitchinson S.L., Stafford K.J., Mellor D.J. (2009). Amelioration of electroencephalographic responses to slaughter by non-penetrating captive bolt stunning after ventral neck incision in halothane anaesthetised calves. N. Z. Vet. J..

[56] Johnson C.B., Sylvester S., Stafford K.J., Mitchinson S., Ward R.N., Mellor D.J. (2009). Effects of age on the electroencephalographic response to castration in lambs anaesthetised using halothane in oxygen from birth to six weeks old. Vet. Anaesth. Analg..

[57] Kells N.J., Beausoleil N.J., Chambers P., Sutherland M.A., Morrison R.S., Johnson C.B. (2017). Electroencephalographic responses of anaesthetized pigs (Sus scrofa) to tail docking using clippers or cautery iron performed at 2 or 20 days of age. Vet. Anaesth. Analg..

[58] Kells N.J., Beausoleil N.J., Sutherland M.A., Johnson C.B. (2018). Electroencephalographic responses of anaesthetised pigs to intraperitoneal injection of sodium pentobarbital. Anim. Welf..

[59] Kells N.J., Beausoleil N.J., Sutherland M.A., Johnson C.B. (2019). Postnatal development of EEG responses to noxious stimulation in pigs (*Sus scrofa*) aged 1–15 days. Anim. Welf..

[60] Diesch T.J., Mellor D.J., Johnson C.B., Lentle R.G. (2010). Developmental changes in the electroencephalogram and responses to a noxious stimulus in anaesthetised tammar wallaby joeys (Macropus eugenii eugenii). Lab. Anim..

[61] Diesch T.J., Mellor D.J., Johnson C.B., Lentle R.G. (2009). Electroencephalographic responses to tail clamping in anaesthetised rat pups. Lab. Anim..

[62] Murrell J.C., Waters D., Mitchinson S.L., Johnson C.B. (2007). Comparative effect of thermal, mechanical and electrical noxious stimuli on the electroencephalogram of the rat. Br. J. Anaesth..

[63] Kongara K., McIlhone A.E., Kells N.J., Johnson C.B. (2014). Electroencephalographic evaluation of decapitation of the anaesthetized rat. Lab. Anim..

[64] Singh P., Kongara K., Harding D., Ward N., Dukkipati V.S.R., Johnson C., Chamber P. (2018). Comparison of electroencephalographic changes in response to acute electrical and thermal stimuli with the tail flick and hot plate test in rats administered with opiorphon. BMC Neurol..

[65] Kongara K., Chambers J.P., Johnson C.B. (2010). 2010 Electroencephalographic responses of tramadol, parecoxib and morphine to acute noxious electrical stimulation in anaesthetised dogs. Res. Vet. Sci..

[66] Fraser D. (2008). Understanding Animal Welfare: The Science in its Cultural Context.

[67] Mellor D.J. (2015). Enhancing animal welfare by creating opportunities for ‘positive affective engagement’. N. Z. Vet. J..

[68] Mellor D.J. (2015). Positive welfare states and promoting environment-focused and animal-to-animal interactive behaviours. N. Z. Vet. J..

[69] Mellor D.J. (2015). Positive animal welfare states and reference standards for welfare assessment. N. Z. Vet. J..

[70] Mellor D.J. (2016). Updating animal welfare thinking: Moving beyond the ‘five freedoms’ towards ‘a life worth living’. Animals.

[71] Mellor D.J. (2017). Operational details of the Five Domains Model and its key applications to the assessment and management of animal welfare. Animals.

[72] Mellor D.J., Beausoleil N.J. (2015). Extending the ‘Five Domains’ model for animal welfare assessment to incorporate positive welfare states. Anim. Welf..

[73] Mellor D.J., Beausoleil N.J., McMillan F.D. (2019). Moving beyond a problem-based focus on poor welfare towards creating opportunities to have positive welfare experiences. Mental Health and Well-Being in Animals.

[74] Molony V., Kent J.E. (1997). Assessment of acute pain in farm animals using behavioural and physiological measurements. J. Anim. Sci..

[75] Taylor P.M., Pascoe P.J., Mama K.R. (2002). Diagnosing and treating pain in the horse: Where are we today?. Vet. Clin. Equine.

[76] Mellor D.J., Stafford K.J., Todd S.E., Lowe T.E., Ward R.N., Gregory N.G., Bruce R.A. (2002). Comparison of cortisol and catecholamine responses to ring castration and tailing of lambs and amputation dehorning of calves. Aust. Vet. J..

[77] Molony V., Kent J.E., McKendrick I.J. (2002). Validation of a method for assessment of an acute pain in lambs. Appl. Anim. Behav. Sci..

[78] Peers A., Mellor D.J., Wintour E.M., Dodic M. (2002). Blood pressure, heart rate, hormonal and other acute responses to rubber ring castration plus tailing of lambs. N. Z. Vet. J..

[79] Rutherford K.M.D. (2002). Assessing pain in animals. Anim. Welf..

[80] Stafford K.J., Mellor D.J., Grandin T. (2015). Painful husbandry procedures in livestock and poultry. Improving Animal Welfare.

[81] Sutherland M.A. (2015). Welfare implications of invasive piglet husbandry procedures, methods of alleviation and alternatives: A review. N. Z. Vet. J..

[82] Sapolsky R.M., Romero L.M., Munck A.U. (2000). How do glucocorticoids influence stress responses? Integrating permissive, suppressive, stimulatory, and preparative actions. Endocrinol. Rev..

[83] Ashley F.H., Waterman-Pearson A.E., Whay H.R. (2005). Behavioural assessment of pain in horses and donkeys: Application to clinical practice and future studies. Equine Vet. J..

[84] Petrie N., Stafford K.J., Mellor D.J., Bruce R.A., Ward R.N. (1995). The behaviour of calves tail docked with a rubber ring used with or without local anaesthetic. Proc. N. Z. Soc. Anim. Prod..

[85] Dinniss A.S., Stafford K.J., Mellor D.J., Bruce R.A., Ward R.N. (1999). The behaviour pattern of lambs after castration using a rubber ring and/or castrating clamp with or without local anaesthetic. N. Z. Vet. J..

[86] Mellor D.J., Stafford K.J. (2004). Physiological and behavioural assessment of pain in ruminants: Principles and caveats. Altern. Lab. Anim..

[87] Stafford K.J., Mellor D.J. (2002). Monitoring pain in animals using behaviour. Proc. N. Z. Soc. Anim. Prod..

[88] Sylvester S.P., Stafford K.J., Mellor D.J., Bruce R.A., Ward R.N. (2004). Behavioural responses of calves to amputation dehorning with and without local anaesthesia. Aust. Vet. J..

[89] Taffarel M.O., Luna S.P.L., de Oliveira F.A., Cardoso G.S., de Moura Alonso J., Pantoja J.C., Brondani J.T., Love E., Taylor P., White K. (2015). Refinement and partial validation of the UNESP-Botucatu multidimensional composite pain scale for assessing postoperative pain in horses. BMC Vet. Res..

[90] Dalla Costa E., Minero M., Lebelt D., Stucke D., Canali E., Leach M.C. (2014). Development of the Horse Grimace Scale (HGS) as a pain assessment tool in horses undergoing routine castration. PLoS ONE.

[91] Dalla Costa E., Stucke D., Dai F., Minero M., Leach M.C., Lebelt D. (2016). Using the horse grimace scale (HGS) to assess pain associated with acute laminitis in horses (*Equus caballus*). Animals.

[92] Wathan J., Burrows A.M., Waller B.M., McComb K. (2015). EquiFACS: The equine facial action coding system. PLoS ONE.

[93] Gleerup K.B., Lindegaard C. (2016). Recognition and quantification of pain in horses: A tutorial review. Equine Vet. Educ..

[94] Gleerup K.B., Forkman B., Lindegaard C., Andersen P.H. (2015). An equine pain face. Vet. Anaesth. Analg..

[95] Machteld C., van Dierendonck M.C., van Loon J.P.A.M. (2016). Monitoring acute equine visceral pain with the equine utrecht university scale for composite pain assessment (EQUUS-COMPASS) and the equine utrecht university scale for facial assessment of pain (EQUUS-FAP): A validation study. Vet. J..

[96] Dyson S., Berger J.M., Ellis A.D., Mullard J. (2017). Can the presence of musculoskeletal pain be determined from the facial expressions of ridden horses (FEReq)?. J. Vet. Behav..

[97] Mullard J., Berger J.M., Ellis A.D., Dyson S. (2017). Development of an ethogram to describe facial expressions in ridden horses (FEReq). J. Vet. Behav..

[98] Torcivia C., McDonell S. (2020). In-person caretaker visits disrupt ongoing discomfort behavior in hospitalized equine orthopedic surgical patients. Animals.

[99] Haggard P., de Boer L. (2014). Oral somatosensory awareness. Neurosci. Biobehav. Rev..

[100] Aspinall V., Cappello M. (2015). Introduction to Veterinary Anatomy and Physiology Textbook.

[101] (2019). Equine Cranial Nerves. Vet Physio Phyle, WordPress.

[102] (2019). Trigeminal Nerve, *Wikipedia*. https://en.wikipedia.org/wiki/Trigeminal_nerve.

[103] (2020). Facial Nerve. Wikipedia.

[104] Bennett D.G. Bits and bitting: Form and function. Proceedings of the 47th Annual Convention of the American Association of Equine Practitioners.

[105] Manfredi J., Clayton H.M., Rosenstein D. (2005). Radiographic study of bit position within the horse’s oral cavity. Equine Comp. Exerc. Physiol..

[106] Benoist C.C., Cross G.H. (2018). A photographic methodology for analyzing bit position under rein tension. J. Equine Vet. Sci..

[107] Mantyh P.W. (2014). The neurobiology of skeletal pain. Eur. J. Neurosci..

[108] Van Lancker S., van den Broeck W., Simoens P. (2007). Incidence and morphology of bone irregularities of the equine interdental space (bars of the mouth). Equine Vet. Educ..

[109] College Physics Tension. Dynamics: Force and Newton’s Laws of Motion.

[110] Clayton H.M., Singleton W.H., Lanovaz J., Cloud G.L. (2003). Measurement of rein tension during horseback riding using strain gage transducers. Exp. Tech..

[111] Clayton H.M., Larson B., Kaiser L.A.J., Lavagnino M. (2011). Length and elasticity of side reins affect rein tension at trot. Vet. J..

[112] Egenvall A., Eisersiö M., Rhodin M., van Weeren R., Roepstorff L. (2015). Rein tension during canter. Comp. Exerc. Physiol..

[113] Egenvall A., Roepstorff L., Eisersiö M., Rhodin M., van Weeren R. (2015). Stride-related rein tension patterns in walk and trot in the ridden horse. Acta Vet. Scand..

[114] Egenvall A., Clayton H.M., Eisersiö M., Roepstorff L., Byström A. (2019). Rein tensions in transitions and halts during equestrian dressage training. Animals.

[115] Piccolo L., Kienapfel K. (2019). Voluntary rein tension in horses when moving unridden in s dressage frame compared with ridden tests in the same horses—A pilot study. Animals.

[116] Heleski C.R., McGreevy P.D., Kaiser L.J., Lavagnino M., Tans E., Bello N., Clayton H.M. (2009). Effects on behaviour and rein tension on horses ridden with or without martingales and rein inserts. Vet. J..

[117] Christensen J.W., Zharkikh T.L., Antoine A., Malmkvist J. (2011). Rein tension acceptance in young horses in a voluntary test situation. Equine Vet. J..

[118] Mellor D.J. Equine Welfare during Exercise: Do We Have a ‘Bit’ of a Problem. PowerPoint Slides Presented at a Professional Development Event, Entitled *Sport Horse Welfare and Social Licence to Operate*, Mounted by Horse South Australia on 13 and 14 February 2018 at Hahndorf, South Australia. https://vetphysiophyle.wordpress.com/2019/04/04/equine-cranial-nerves/.

[119] Bendrey R. (2007). New methods for the identification of evidence for bitting on horse remains from archaeological sites. J. Archaeol. Sci..

[120] Mata F., Johnson C., Bishop C. (2015). A cross-sectional epidemiological study of prevalence and severity of bit-induced oral trauma in polo ponies and race horses. J. Appl. Anim. Welf. Sci..

[121] Tremaine W.H. (1998). Management of equine mandibular injuries. Equine Vet. Educ..

[122] Johnson T.J. (2002). Surgical removal of mandibular periostitis bone spurs caused by bit damage. Proc. Am. Assoc. Equine Pract..

[123] Johnson J., Porter M. (2006). Dental conditions affecting the mature performance horse (5–15 years). Proc. Am. Assoc. Equine Pract..

[124] Tuomola K., Maki-Kinnia N., Kujala-Wirth M., Mykkänen A., Valros A. (2019). Oral lesions in the bit area in finnish trotters after a race: Lesion evaluation, scoring and occurrence. Front. Vet. Sci..

[125] Björnsdóttir S., Frey R., Kristjansson T., Lundström T. (2014). Bit-related lesions in Icelandic competition horses. Acta Vet. Scand..

[126] Byers M.R., Närhi M.V. (1999). Dental injury models: Experimental tools for understanding neuroinflammatory interactions and polymodal nociceptor functions. Crit. Rev. Oral Biol. Med..

[127] Equus 2019. Tongue Injuries: Wounds to Your Horse’s Tongue Can Easily Go Unnoticed—But That Doesn’t Mean They Can be Ignored. https://equusmagazine.com/horse-care/tongue-injuries-12258.

[128] Pigg M., Svensson P., List T. (2011). Orofacial thermal thresholds: Time-dependent variability and influence of spatial summation and test site. J. Orofac. Pain.

[129] Tell A., Egenvall A., Lundstrom T., Wattle O. (2008). The prevalence of oral ulceration in Swedish horses when ridden with bit and bridle and when unridden. Vet. J..

[130] Uldahl M., Clayton H. (2018). Lesions associated with the use of bits, nosebands, spurs and whips in Danish competition horses. Equine Vet. Educ..

[131] Rezaian M. (2006). Absence of hyaline cartilage in the tongue of ‘Caspian miniature horse’. Anat. Histol. Embryol..

[132] Engelke E., Gasse H. (2003). An anatomical study of the rostral part of the equine oral cavity with respect to position and size of a snaffle bit. Equine Vet. Educ..

[133] Manfredi J.M., Rosenstein D., Lanovaz J.L., Nauwelaerts S., Clayton H.M. (2010). Fluoroscopic study of oral behaviours in response to the presence of a bit and the effects of rein tension. Comp. Exerc. Physiol..

[134] Findley J.A., Sealy H., Franklin S.H. (2016). Factors associated with tongue tie use in Australian Standardbred racehorses. Equine Vet. J..

[135] Barakzai S.Z., Finnegan C., Dixon P.M., Hillyer M.H., Boden L.A. (2009). Use of tongue ties in thoroughbred racehorses in the United Kingdom, and its association with surgery for dorsal displacement of the soft palate. Vet. Rec..

[136] Porter D., Caraguel C., Noschka E., Samantha Franklin S. Tongue-tie use in Australian Thoroughbred horses over a 5-year period (2009–2013). Proceedings of the World Equine Airway Symposium.

[137] Franklin S.H., Naylor J.R., Lane J.G. (2002). The effect of a tongue-tie in horses with dorsal displacement of the soft palate. Equine Vet. J..

[138] Vandermark S., Wilkins C. (2019). Tongue Ties: Trying to See the Whole Picture. Horses and People.

[139] Franklin S., McGreevy P. (2018). Over 20% of Australian Horses Race with Their Tongues Tied to Their Lower Jaw. The Conversation.

[140] Marsh L., McGreevy P., Hazel S., Santos L., Herbart M., Franklin S. (2019). The effect of tongue-tie application on stress responses in resting horses. BioRxiv.

[141] Dyson S., Berger J.M., Ellis A.D., Mullard J. (2018). Development of an ethogram for a pain scoring system in ridden horses and its application to determine the presence of musculoskeletal pain. J. Vet. Behav..

[142] Muir W. Recognizing and Treating Pain in Horses, *Veterian Key* 2016. https://veteriankey.com/recognizing-and-treating-pain-in-horses/.

[143] Mair T., Lane G. (1990). Head shaking in horses. In Practice.

[144] Newton S.A., Knottenbelt D.C., Eldridge P.R. (2000). Headshaking in horses: Possible aetiopathogenesis suggested by the results of diagnostic tests and several treatment regimes used in 20 cases. Equine Vet. J..

[145] Roberts V. (2019). Trigeminal-mediated headshaking in horses: Prevalence, impact, and management strategies. Vet. Med. Res. Rep..

[146] Cook W.R. (1999). Pathophysiology of bit control in the horse. J. Equine Vet. Sci..

[147] Cook W.R., Mills D.S. (2009). Preliminary study of jointed snaffle vs. crossunder bitless bridles: Quantified comparison of behaviour in four horses. Equine Vet. J..

[148] Hanson F., Cook R. (2015). The Bedouin bridle rediscovered: A welfare, safety and performance enhancer. Horse’s Hoof.

[149] Carey C. The impact of bitless vs. bitted bridles on the therapeutic riding horse research project. Presented at the Horses in Education and Therapy International Conference on Therapeutic Riding.

[150] Carey C., Hayes-Moriarty S., Brennan R. The impact of bitted and bitless bridles on the therapeutic riding horse. Proceedings of the 12th International Equitation Science Conference on Understanding Horses to Improve Training and Performance.

[151] Carey C., Brennan R., Hayes-Moriarty S. (2020). Further Study on the Impact of Bitted vs. Bitless Bridles for Therapeutic Riding Equines. Festiina Lente Newsletter.

[152] Polito R., Minero M., Canali E., Verga M. (2007). A pilot study on Yearlings’ reactions to handling in relation to the training method. Anthrozöos.

[153] Visser E.K., Van Dierendonck M., Ellis A.D., Rijksen C., Van Reenen C.G. (2009). A comparison of sympathetic and conventional training methods on responses to initial horse training. Vet. J..

[154] Von Borstel U.U., Duncan I.J.H., Shoveller A.K., Merkies K., Linda Jane Keeling L.J., Millman S.T. (2009). Impact of riding in a coercively obtained Rollkur posture on welfare and fear of performance horses. Appl. Anim. Behav. Sci..

[155] McLean A.N., McGreevy P.D. (2010). Horse-training techniques that may defy the principles of learning theory and compromise welfare. J. Vet. Behav..

[156] Hall C., Kay R., Yarnell K. (2014). Assessing ridden horse behavior: Professional judgment and physiological measures. J. Vet. Behav..

[157] Górecka-Bruzda A., Kosinska I., Jaworski Z., Tadeusz Jezierski T., Murphy J. (2015). Conflict behavior in elite show jumping and dressage horses. J. Vet. Behav..

[158] Clayton H. (2019). Are Horses Stressed When Bitted for the First Time?. Eurodressage.

[159] Fraser A.F. (1992). The Behaviour of the Horse.

[160] Ransom J.I., Cade B.S. (2009). Quantifying equid behavior: A research ethogram for free-roaming feral horses. U.S. Geological Survey Techniques and Methods Report 2-A9.

[161] King M. Bitless: A New Breed of Bridle. The Horse, August 2007. https://thehorse.com/124806/bitless-a-new-breed-of-bridle/.

[162] Hanson F. (2019). A positive reinforcement rein: Rule-changer and game-changer for horsemanship?. Horse’s Hoof.

[163] Ambrosiano N. (2017). All about Bitless Bridles for Your Horse: Bit-Free Headgear Is Sometimes the Answer for Sensitive Horses or Tough Training Problems. Equus.

[164] (2019). Bitless Bridle. Wikipedia.

[165] Bosal, *Wikipedia* 2018. https://en.wikipedia.org/wiki/Bosal.

[166] Hackamore, *Wikipedia* 2020. https://en.wikipedia.org/wiki/Hackamore.

[167] Ramey D.W., McIlwraith C.W., Rollin B.E. (2011). A historical survey of human-equine interactions. Equine Welfare.

[168] Whay H.R., Main D.C.J., Green L.E., Webster A.J.F. Farmer perception of lameness prevalence. Proceedings of the 12th International Symposium on Lameness in Ruminants.

[169] Barker Z.E., Leach K.A., Whay H.R., Bell N.J., Main D.C.J. (2010). Assessment of lameness prevalence and associated risk factors in dairy herds in England and Wales. J. Dairy Sci..

[170] Horseman S.V., Roe E.J., Huxley J.N., Bell N.J., Mason C.S., Whay H.R. (2014). The use of in-depth interviews to understand the process of treating lame dairy cows from the farmers’ perspective. Anim. Welf..

[171] Beausoleil N.J., Mellor D.J. (2015). Introducing breathlessness as an animal welfare issue. N. Z. Vet. J..

[172] (2017). Poiseuille’s Law: IV Fluids, *Open Anaesthesia*. https://www.openanesthesia.org/poiseuilles_law_iv_fluids/.

[173] Cook W.R. (2014). A hypothetical, aetiological relationship between the horse’s bit, nasopharyngeal oedema and negative pressure pulmonary oedema. Equine Vet. Educ..

[174] Cook W.R. (2016). Hypothesis article: Bit-induced asphyxia in the racehorse as a cause of sudden death. Equine Vet. J..

[175] Dixon P.M., Railton D.I., McGorum B.C. (1993). Temporary bilateral laryngeal paralysis in a horse associated with general anaesthesia and post anaesthetic myositis. Vet. Rec..

[176] Kollias-Baker C.A., Pipers F.S., Heard D., Seeherman H. (1993). Pulmonary edema associated with transient airway obstruction in three horses. J. Am. Vet. Med Assoc..

[177] Tute A.S., Wilkins P.A., Gleed R.D., Credille K.M., Murphy D.J., Ducharme N.G. (1996). Negative pressure pulmonary edema as a post-anesthetic complication associated with upper airway obstruction in a horse. Vet. Surg..

[178] McGreevy P.D., Harman A., McLean A., Hawson L. (2010). Over-flexing the horse’s neck: A modern equestrian obsession?. J. Vet. Behav..

[179] McLean A.N., McGreevy P.D. (2010). Ethical equitation: Capping the price horses pay for human glory. J. Vet. Behav..

[180] McGreevy P.D., McLean A.N., Warren-Smith A.K., Waran N., Goodwin D. (2005). Defining the terms and processes associated with equitation. Proceedings of the 1st International Equitation Science Symposium 2005.

[181] McGreevy P., Warren-Smith A., Guisard Y. (2012). The effect of double bridles and jaw-clamping crank nosebands on facial cutaneous and ocular temperature in horses. J. Vet. Behav. Clin. Appl. Res..

[182] Fenner K., Yoon S., White P., Starling M., McGreevy P. (2016). The effect of noseband tightening on horses’ behavior, eye temperature, and cardiac responses. PLoS ONE.

[183] Doherty O., Conway T., Conway R., Murray G., Casey V. (2017). An objective measure of noseband tightness and its measurement using a novel digital tightness gauge. PLoS ONE.

[184] Global Dressage Forum (2011). Bitless or Not: It about Having the Choice. Eurodressage.

[185] North American Dressage Association 2020. https://www.northamericanwesterndressage.com.

[186] Western Dressage Association of America 2020. https://westerndressageassociation.org.

